# Effect of the Topology on Wetting and Drying of Hydrophobic Porous Materials

**DOI:** 10.1021/acsami.2c06039

**Published:** 2022-06-22

**Authors:** Yuriy G. Bushuev, Yaroslav Grosu, Mirosław A. Chorążewski, Simone Meloni

**Affiliations:** † Institute of Chemistry, University of Silesia in Katowice, Szkolna 9 street, 40-006 Katowice, Poland; ‡ Centre for Cooperative Research on Alternative Energies (CIC energiGUNE), Basque Research and Technology Alliance (BRTA), Alava Technology Park, Albert Einstein 48, 01510 Vitoria-Gasteiz, Spain; § Dipartimento di Scienze Chimiche, Farmaceutiche ed Agrarie (DOCPAS), Università degli Studi di Ferrara (Unife), Via Luigi Borsari 46, I-44121 Ferrara, Italy

**Keywords:** nanoporous materials, hydrophobic nanotubes, pure silica zeolites, intrusion/extrusion, solid−liquid interface

## Abstract

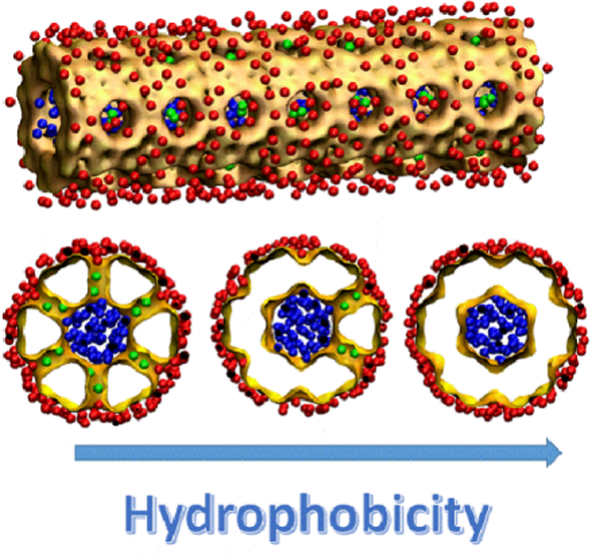

Establishing molecular mechanisms of wetting and drying of hydrophobic porous materials is a general problem for science and technology within the subcategories of the theory of liquids, chromatography, nanofluidics, energy storage, recuperation, and dissipation. In this article, we demonstrate a new way to tackle this problem by exploring the effect of the topology of pure silica nanoparticles, nanotubes, and zeolites. Using molecular dynamics simulations, we show how secondary porosity promotes the intrusion of water into micropores and affects the hydrophobicity of materials. It is demonstrated herein that for nano-objects, the hydrophobicity can be controlled by changing the ratio of open to closed nanometer-sized lateral pores. This effect can be exploited to produce new materials for practical applications when the hydrophobicity needs to be regulated without significantly changing the chemistry or structure of the materials. Based on these simulations and theoretical considerations, for pure silica zeolites, we examined and then classified the experimental database of intrusion pressures, thus leading to the prediction of any zeolite’s intrusion pressure. We show a correlation between the intrusion pressure and the ratio of the accessible pore surface area to total pore volume. The correlation is valid for some zeolites and mesoporous materials. It can facilitate choosing prospective candidates for further investigation and possible exploitation, especially for energy storage, recuperation, and dissipation.

## Introduction

Zeolites, metal–organic frameworks (MOFs), silica nanotubes, and mesoporous amorphous silica are natural and synthetic porous materials intensively used for both fundamental studies and industrial applications.^1−4^ Catalysis, purification, and separation of liquids and gases, energy storage, and conversion are examples of their applications.^5−7^ Moreover, microporosity plays a crucial role in the vital functions of living organisms.^8,9^

Zeolites, MOFs, and zeolitic imidazolate frameworks (ZIFs) are crystalline microporous materials with different topologies. In this case, a porous system is ordered, and the pore size distribution is narrow. A three-letter code designates the topology of zeolites.^10^ Some ZIFs and zeolites have the same topology, but the geometrical sizes of pores and water interactions with pore walls are different and depend on the chemical composition.^1,2^ These microporous materials have a large surface area, high pore volume, and outstanding adsorption performance. They are exploited for adsorptive removal/separation and purification of gases and extraction of various hazardous liquids from aqueous systems.^11,12^

The most common type of porous materials is the ones possessing a disordered or random porous network. For example, mesoporous amorphous silicas have attracted increasing attention as catalysts,^13^ drug delivery vehicles,^14^ and solid components of heterogeneous lyophobic systems (HLSs).^15−17^ The hydrophobicity of the materials is varied in a broad range depending on their functionalization.^18,19^ Hierarchically structured porous materials^20^ templating mesoporous zeolites^21^ have micro- and mesoporosity. It was shown^22,23^ that depending on the temperature, the topology of porous materials can be changed by selecting the synthesis conditions.

Many methods are used to synthesize silica nanoscale materials.^24^ Silica nanotubes of different sizes are fabricated. Their inner and outer surfaces can be functionalized using simple silane chemistry with various chemical functional groups.^25−27^ Nanotubes with hydrophilic outer surfaces and hydrophobic inner surfaces are ideal for extracting lipophilic molecules from aqueous solutions. Functionalized silica nanotubes can be used for bioseparation and biocatalysis. Another application of mesoporous nanoparticles is construction of drug delivery vehicles.^14^ Thus, controlling the hydrophobicity of modified silica nanotubes extends their applicability.

Some promising technologies for adsorption, storage, and conversion of mechanical,^28^ thermal,^7,29^ and electrical^30^ energy are based on HLSs, consisting of porous materials and a nonwetting liquid. Water, aqueous solutions, and hydrophobic porous materials represent a particular class of HLSs. Water intrudes into hydrophobic porous materials only at high pressure.^16^ Extrusion occurs at a pressure similar to or lower than intrusion pressure or may not occur due to metastabilities. These systems are called molecular springs, shock absorbers, and bumpers, respectively.^31^ Actual materials have different pore sizes and topologies and a degree of hydrophobicity. These are the main parameters controlling their wetting and drying. The influence of the topology of the pore on the intrusion/extrusion pressure is still not well defined.

Among well-known and widely used controlling parameters such as the chemical composition of a material, geometry of pores, and temperature, less attention was paid to the topology of a pore system as one of the key factors determining fluid transport through the porous material, energetic capacity of HLSs. Only limited experimental data about energetic performance and intrusion/extrusion pressure for water and aqueous solutions are available.^15,31,32^

According to the Laplace–Washburn equation, the capillary pressure (*P*_c_) depends on the liquid–vapor (γ_lv_) surface tension, contact angle (θ), solid–liquid (γ_sl_) and solid–vapor (γ_sv_) surface tensions, and pore radius (*r*)
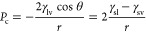
1Intrusion occurs when hydrostatic pressure becomes equal to the capillary pressure. However, in the case of microporosity, when the size of pores is comparable with the size of molecules, the predictions according to eq 1 may be incorrect. The structure of water and intermolecular interactions under nanoconfinement differ from bulk liquid. For pure silica zeolites (PSZs), intrusion pressure and reverse radius correlate poorly.^15,31,32^

Despite intensive investigations,^2,33,34^ peculiarities of intermolecular interactions, molecular mechanisms of processes, and the structure of liquids under nanoconfinement are not well established. The prediction of intrusion pressure or the hydrophobicity of the materials for HLS is a very actual task.

Recently, we published the results of computer simulations of ITT and MFI-type PSZs and corresponding to them zeolite nanoparticles with the modified topology of frameworks immersed in water.^35^ It was demonstrated that it is possible to control water intrusion pressure by tuning the topology of nanoparticles.

The present paper’s goal is to establish a molecular mechanism of topological tuning of the hydrophobicity of porous materials. Our objects of investigation are pure silica nanoparticles and nanotubes tailored from the ITT-type zeolite crystal. The system of zeolite micropores consists of straight channels with nanometric aperture interconnected by subnanometric pores. We investigate the role of these lateral pores, the secondary porosity, in wetting/drying processes. Based on intrusion/extrusion isotherms and kinetic curves, we show how the closing of lateral pores affects pressures and how water in the pores stabilizes water in the channels. Simple theoretical considerations are provided to support and rationalize computational results. Finally, based on an established correlation between intrusion pressure and a ratio of accessible surface area to pore volume, we analyze existing experimental data for zeolites and predict the pressure for other zeolites. Controlling the intrusion/extrusion pressure by changing the topology of pores opens a new path for the design, fabrication, and exploitation of porous materials with regulated hydrophobicity.

## Results and Discussion

### Models of Nano-objects

Among topologies presented in the Database of Zeolite Structures,^10^ the ITT-type framework with extra-large 18MR channels and a pore aperture of 15.3 Å is the most suitable for our purpose. Each 18MR channel has six rows of 10MR lateral pores along the surface with an aperture of 7.8 Å. The 10MR pores are ubiquitous in zeolite topologies, especially among zeolites widely used for practical applications.^36^ The objects with approximately cylindrical channels and small lateral pores on their surface can highlight the role of secondary porosity.

The silicogermanate ITQ-33 zeolite has the ITT topology.^37^ However, experimental data about water intrusion into ITQ-33 are absent. We use ITT-type nanoparticles as an artificial system whose topology can be modified easily by blocking 10MR pores. It transforms the interconnected three-dimensional (3D) network of pores into a one-dimensional (1D) system of isolated channels. The hydrophobicity of two nanoparticles immersed in water was investigated: with open and closed 10MR pores. In the following, the original and modified ITT particles, where all 10MR pores are blocked, are termed “0 closed rows” and “6 closed rows” nanoparticles, respectively. Multiwall silica nanotubes were tailored from the ITT crystal too.

According to Kiselev’s approach,^38^ we used rigid frameworks and neglected electrostatic interactions for Si and O atoms. The effect of framework flexibility and partial charges on the atoms is discussed in the previous article.^35^

Models of porous nanomaterials with closed 10MR pores are designed to highlight the role of topological tuning and demonstrate how the topology affects the hydrophobicity. To modify the solids, we closed 10MR pores by bridging oxygen atoms lying at the opposite sides of the 10MR pores by rigid −Si–O–Si– chains, making them impenetrable for water. Thus, the lateral pores were closed by the same atoms that cover the inner surface of the tubes. Due to the tube rigidity, we do not consider the valence of bridging atoms and the direction of bonds.

**Figure 1:**
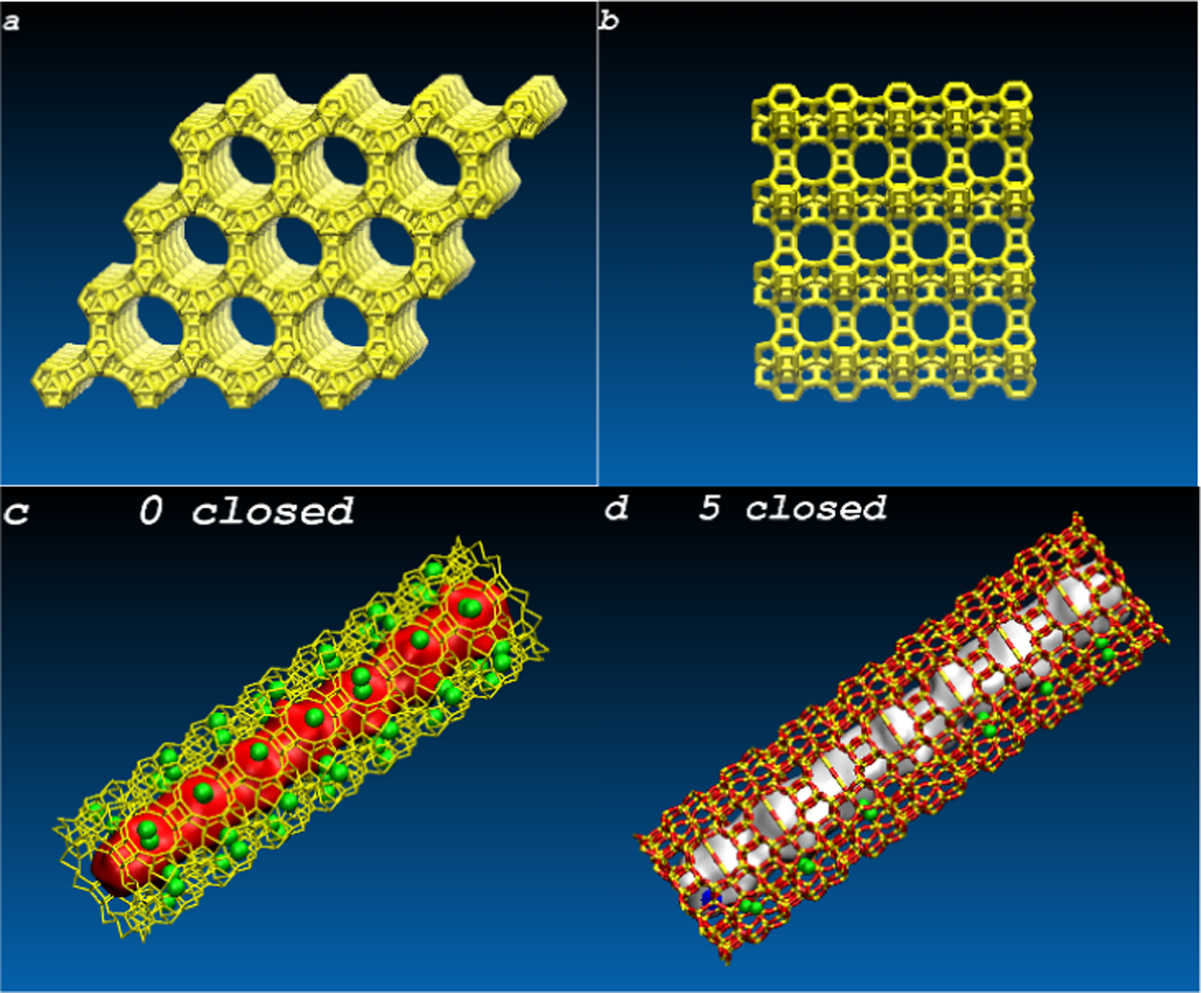
(a, b) Nanoparticle tailored from the ITT framework. Each hexagonal 18MR channel in the bulk of the crystal connects with six adjoining channels through 10MR pores. (c, d) Two nanotubes with all open 10MR pores and 5 closed rows of the pores. For clarity, water in the nanotubes is shown by solid surfaces. The green balls represent water in 10MR pores. −Si–O–Si– chains close 10MR pores (d). Zeolitic oxygen atoms are not shown in (a–c).

Two particles with open and closed 10MR pores and four nanotubes were fabricated, designated as 6, 5, 4 closed rows and open (0 closed rows), depending on the number of closed pore rows along tube surfaces. For illustration, the ITT nanoparticle with open pores and two nanotubes are presented in Figure 1. The nano-objects were immersed in water. Indifferent from particles, all open pores of tubes were in contact with the surrounding water. Moreover, this water interacts with water in the tubes through the thin wall. The length of tubes was higher than that of channels in the particles, which demands a longer intrusion/extrusion time, making kinetics more representative. Molecular dynamics simulations of the systems were performed at *T* = 300 K and a set of hydrostatic pressures.

We selected ITT-type zeolitic nano-objects as models because the results can be related to a broad range of materials, including microporous PSZs and mesoporous amorphous silica. Functionalization of silica materials by organic compounds affects pore sizes and their hydrophobicity. The interaction of the covered surface long alkyl chains with water differs from silica–water interactions. To make nano-objects highly hydrophobic, we exploited the advantage of framework rigidity. The neglection of silica–water electrostatic interactions increases the hydrophobicity. Thus, the nanoparticles mimic grafted mesoporous silica materials.

### Intrusion/Extrusion Isotherms

According to eq 1, the intrusion pressure corresponds to the average hydrophobicity of materials traditionally defined by the contact angle. Water in micropores forms clusters—discrete hydrogen-bonded assemblies of molecules. Parameters such as surface tension or contact angle lose physical meaning in the case of nanoconfinement. It is possible to calculate only the energy of water–water and water–solid interactions. Depending on the process direction, the pressures corresponding to half of the pore loading are intrusion (*P*_intr_) and extrusion (*P*_extr_) pressures. *P*_intr_ is the macroscopic parameter characterizing the hydrophobicity of a microporous material.

**Figure 2:**
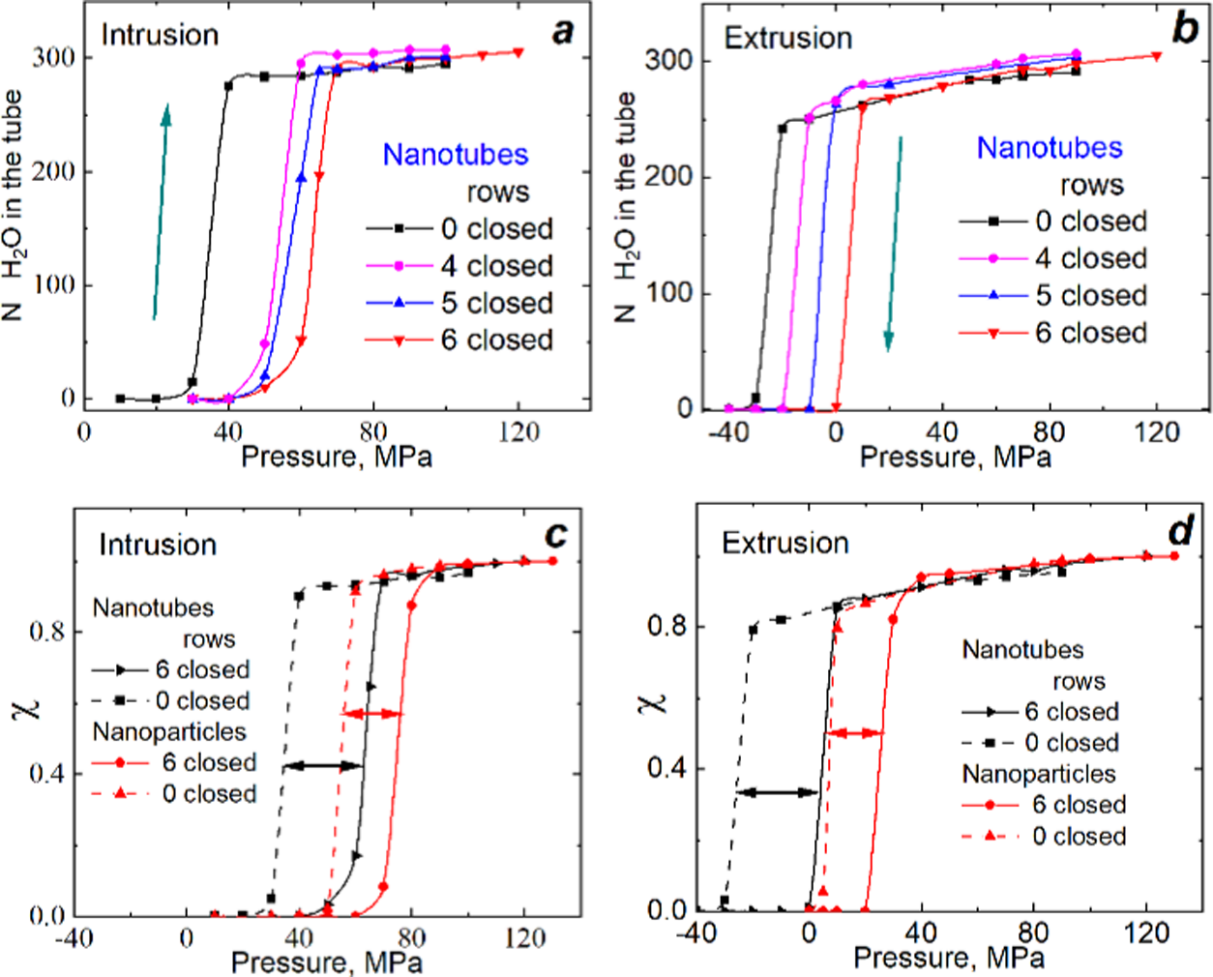
Intrusion (a) and extrusion (b) isotherms for nanotubes. (c, d) Intrusion/extrusion isotherms for nanotubes and corresponding nanoparticles presented as fractional loadings vs pressure.

We observed an abrupt transition between empty and filled states of nano-objects with increasing pressure, resembling a phase transition. The calculated isotherms for nanotubes are shown in Figure 2a,b. Intrusion and extrusion pressures and therefore the hydrophobicity increase with the number of closed pores on the surfaces of tubes. Only one system where all pores are closed demonstrates the shock absorption behavior. Other systems are bumpers because extrusion occurs at negative pressures.

In some cases, before extrusion, clusters occur in a metastable state.^39,40^ The process lasts seconds or even hours, making the measurement of *P*_extr_ difficult.^41^ Meanwhile, in computer simulations, we can observe only several nanoseconds of the evolution of a system. As a result, the calculated extrusion pressure may be less than the measured one in actual experiments. We have not calculated the free energy profile for our systems and suppose that extrusion from actual ITT zeolite may be observed at higher pressure. Calculations for microporous systems with the molecular spring behavior demonstrate the absence of intrusion/extrusion hysteresis and sometimes even the absence of the metastable state.^15,35,42^

To compare intrusion into nanoparticles and nanotubes, we calculated fractional loadings, χ = *N*/*N*_max_, where *N*_max_ is the maximum number of water molecules in a nano-object. Intrusion and extrusion isotherms presented in Figure 2c,d demonstrate how *P*_intr_ depends on the secondary porosity of particles and tubes, showing larger hydrophobicity of particles. Due to long-range electrostatic interactions, water molecules in nanotubes interact with bulk water through the layers of Si and O atoms. In nanoparticles, the water–water interaction is stronger because a larger volume is occupied by electrically neutral atoms, as is adopted in our force field. Weaker water–silica interactions substitute long-range water–water ones. It explains the larger hydrophobicity of the nanoparticles with respect to nanotubes.

Thus, the intrusion pressure increases with the number of closed rows of pores on the surface of channels. This unexpected result contradicts eq 1 because comparing the apertures of pores, the intrusion pressure into 10MR pores must be twice as large as that into 18MR channels. According to macroscopic theory, the narrow pores must be empty until the pressure reaches the value of the second intrusion pressure. The extrusion pressure increases with the number of closed pores too. In all cases, it is lower than that of intrusion.

The nanoparticle presented in Figure 1a consists of a 3 × 3 grid of 18MR channels. Depending on their positions, the channels have a different number of 10MR pores connecting them with bulk water. Based on the previous analysis of intrusion kinetics for nanoparticles, we concluded that intrusion (crystal wetting) starts from the side channels and propagates into the bulk of the crystal.^35^ In the next section, we pay more attention to intrusion into nanotubes.

### Kinetics of Intrusion

We counted the number of water molecules (*N*) in nanoparticle channels or nanotubes at time *t* to characterize the intrusion processes. The second characteristic is the time evolution of the ratio *N*_t_/*N*_p_, where *N*_t_ and *N*_p_ are the number of molecules in the tube and 10MR pores, respectively.

**Figure 3:**
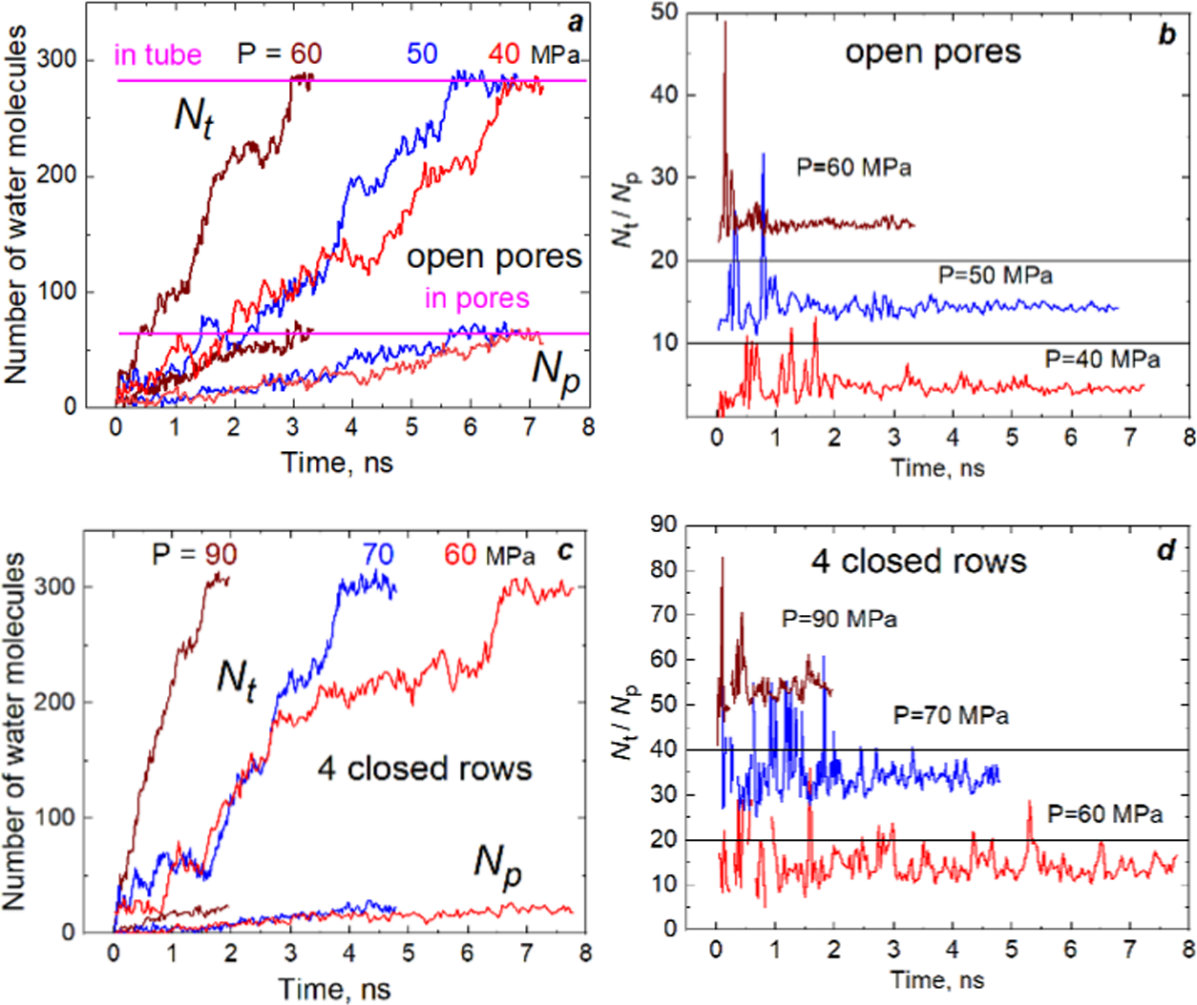
(a, c) Time evolution of the number of water molecules intruding into the “0 closed rows” and “4 closed rows” nanotubes. (b, d) Ratio of the number of molecules in the tubes to that in 10MR pores. Plots for *P* = 50 and 60 MPa (b) and *P* = 70 and 90 MPa (d) are shifted along the *y*-axis for clarity.

The previous article discussed the kinetic curves for nanoparticles;^35^ those for nanotubes curves are presented in Figure 3. The rate of filling depends on the proximity of applied pressure to *P*_intr_. The process is fast at high pressure. The curves demonstrate that some 10MR pores are wet even when the tube is empty. This observation contradicts expectations based on eq 1. During an intrusion, both numbers of water in the tube (*N*_t_) and pores (*N*_p_) increase with time, but their ratio rapidly reaches plateau. This ratio does not depend on pressure, and it equals 4.6 ± 0.4 for the tube with open pores. If only two rows of pores are open, the ratio is three times larger (14.4 ± 2.8), as shown in Figure 3d. For the “5 closed rows” tube, the ratio is ca. 27.1.

Figures 2 and 3 demonstrate that water in pores stabilizes water clusters in tubes—the more the open pores, the lower the intrusion pressure. Movie S1 (see Figure S3) details a molecular mechanism of water intrusion into the nanotube with open pores. Here, an anchoring role of water molecules in pores is visible. Even when the tube is empty, the molecules appear in pores for a short time, but they stay there much longer if a growing water cluster wets the pores. The amount of water in the tube and lateral pores grows simultaneously.

**Figure 4:**
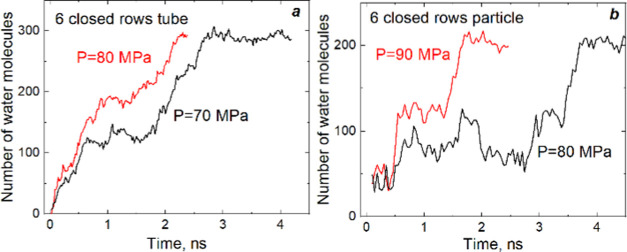
Time evolution of the number of water molecules in the tube (a) and the particle (b) with all-closed pores.

The step-by-step clusters grown are observed for the tube and particle with all-closed lateral pores, as illustrated in Figure 4. The growth of water clusters stops, and they can even shrink in size for a while. After that, the forward motion is restored. Water clusters are unstable at a pressure near *P*_intr_. Fast and slow variations of their sizes are observed, as shown in Movie S2. In the intermediate cases, when parts of pores are closed, this mechanism appears not so sharp (Figure 3c). Thus, again, it highlights the stabilizing role of water in 10MR pores.

### Kinetics of Extrusion

**Figure 5:**
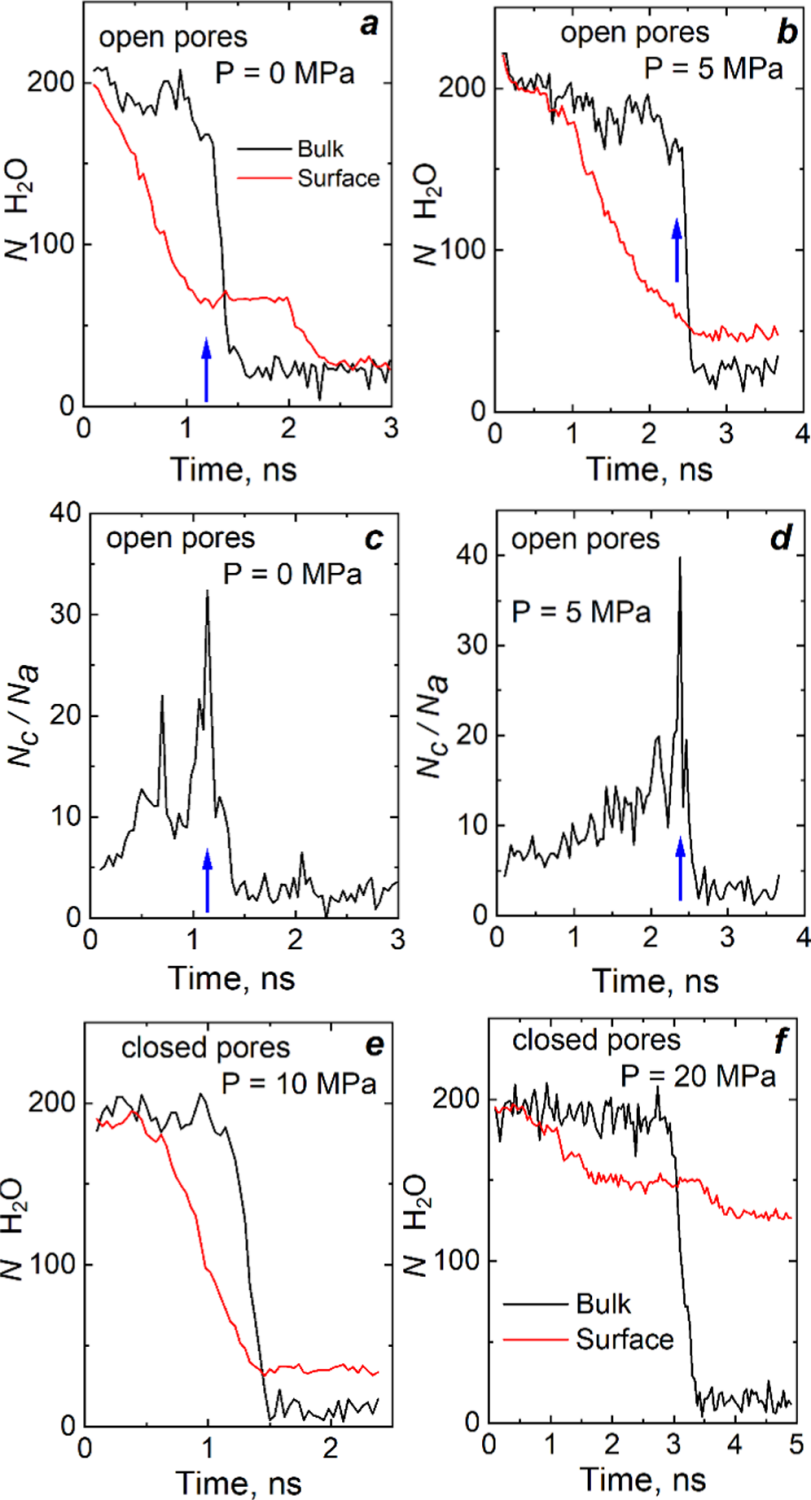
Kinetics of water extrusion from channels: nanoparticle with open (a, b) and closed (e, f) pores. Number of molecules vs time: in the bulk 18MR (black line) and surface (red line) channels. (c, d) Ratio of the number of molecules in the central channel (*N*_c_) to that (*N*_a_) in adjoining 10MR pores. The arrows indicate the starting time of extrusion from the bulk channel.

We observed several drying processes of the wetted nano-objects, starting simulations at a pressure lower than *P*_extr_ from different initial configurations. Kinetic curves for extrusion presented in Figures 5 and S1 demonstrate a significant difference with the process of intrusion. The nanoparticles have nine 18MR channels, but only the central channel has no direct connection through 10MR pores with water surrounding a particle. Water is expelled from particle channels spontaneously. The plateau in Figure 5a corresponds to configurations with seven empty channels, including the central one (bulk).

Drying the adjoining 10MR pores antecedes fast transition to an empty state of the channel, appearing as sharp peaks in Figure 5c,d. Before expulsion from the channel, the water cluster is thinning in some regions till the molecular bridge connecting water is formed. The illustrating snapshots are presented in Figure S2. After losing connectivity, two cylindrical water clusters move to the channel’s mouths at high speed. The calculation shows a linear velocity of water molecules of about 100 m/s. The process of water expulsion from the pore resembles a breaking string under tension. The rate of channel dewetting depends on the state of adjacent channels. Each empty adjoining channel provokes drying of the central one. Movie S3 illustrates the process of extrusion from the nanotube with open pores. We observed similar processes for nanoparticles.

Two plots for extrusion from nanoparticles with open and closed pores are shown in Figure 5e,f. The closer the pressure to *P*_extr_, the longer the extrusion process, but we see the abrupt water expulsion again from each channel. There are no supporting cluster stability water molecules in the 10MR pores, and extrusion spontaneously occurs at a higher pressure than in the case of the nanoparticle with open pores.

**Figure 6:**
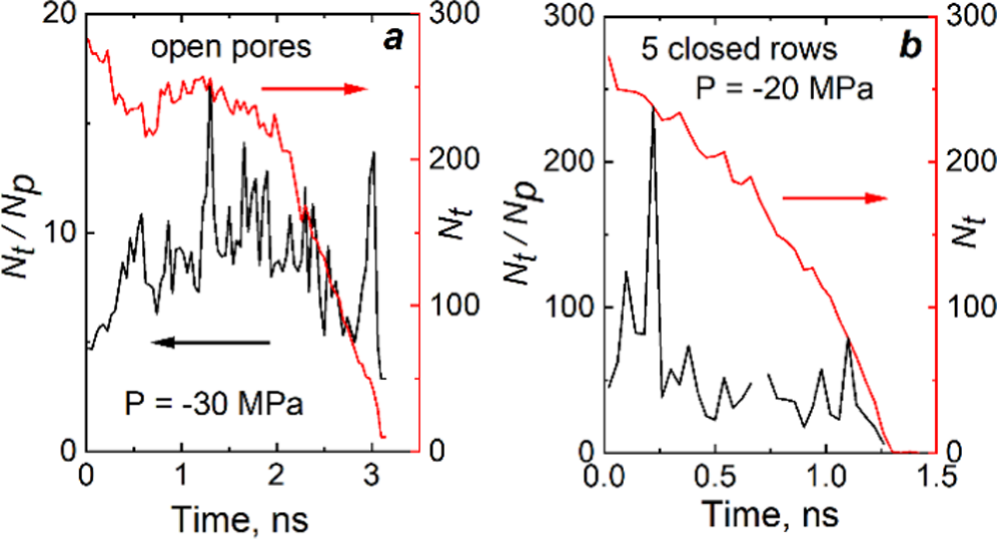
Kinetics of water extrusion from nanotubes: (a) for the tube with open pores and (b) for the tube with five closed rows of pores. *N*_t_ and *N*_p_ are the numbers of water molecules in the tube and pores, respectively.

The extrusion processes from two nanotubes are illustrated in Figure 6. Beginning from time 0 ns, dewetting of narrow pores initiates. For both tubes, the *N*_t_/*N*_p_ ratio grows, fluctuations of these ratios increase, and water loses connectivity and begins to expel. However, extrusion is slower when compared to the bulk channel of nanoparticles due to spontaneous wetting of 10MR pores in tubes by the surrounding water. Comparing extrusion from two nano-objects, we make the same conclusion as in the previous section—water molecules in the pores stabilize the cluster playing the anchoring role. In closed pore windows, intrusion–extrusion occurs at higher pressure.

### Theoretical Consideration

Calculation of the free energy of water in a hydrophobic channel is a complex task.^43^ Classical macroscopic considerations are not fully applicable for narrow micropores. The front of propagating water consists of just a few molecules already penetrated in the 18MR channel. Strictly speaking, macroscopic concepts, such as surface and line tension or a contact angle, do not apply at these scales, preventing a rigorous wetting theory application.^44,45^ Nevertheless, the heuristic use of macroscopic concepts helps us to rationalize present results and compare them with literature data to identify design principles to control hydrophobicity. In particular, we focus on the interaction of water with the nanotube walls decorated by holes that can be hydrophobic or hydrophilic depending on their wetting.

At equilibrium, the Gibbs free energies for water in the bulk phase and the pore are equal, *G*_ww_ = *G*_wp_. For the same amount of water in the bulk and pores:
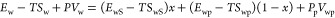
2where *E*_w_, *E*_wS_, and *E*_wp_ are the energies of water in the bulk phase, at pore surfaces, and far from interfaces, respectively. We use the same notation for entropies *S*_*i*_. *V*_w_ and *V*_wp_ are the water volumes in the bulk phase and the pore, respectively. *P* is the intrusion pressure, and *P*_p_ is the pressure in the pore; *x* is the mole fraction of water at interfaces. Taking into account simplifications *P* ≫ *P*_p_ and *V* = *V*_w_ ≈ *V*_wp_, we can rewrite eq 2
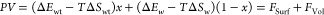
3where Δ*E*_*i*_ and Δ*S*_*i*_ are excess energies and entropies of water in the pore, respectively, and *F*_Surf_ and *F*_Vol_ are the excess free energies for water molecules at the surface and pore volume, respectively.

Here, we adopt an approach for calculating the energetic cost of water penetration in a hydrophobic channel based on a simplified representation of interatomic interactions. In our simple model, instead of the hexagonal 18MR nanotube, we consider a cylindrical tube with holes on the surface, whose number, size, and positions correspond to 10MR pores in the ITT nanotube. Our model assumes that the front of intruded water is flat and sharp, and its profile and (infinitesimal) thickness do not change along the process (Figure S4).

First, we focus on the interface excess free energy, which we represent as the sum of four terms corresponding to water in contact with the solid inner surface of the pore, with wet or dry side pores, and vapor
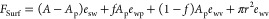
4where *e*_sw_, *e*_wp_, and *e*_wv_ are the excess surface free energies of solid–water, water–wetted pore, and water–vapor, respectively; *f* is the fraction of wetted pores; *A* and *A*_p_ are the areas of the cylinder wall and side pores, respectively; and *r* is the radius of the cylinder. Thus, taking into account the constant values of the last term, we can simplify eq 4
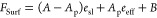
5where *e*_eff_ is the effective free energy of water in contact with partially filled side pores per area of pores and *B* is the positive constant.

For wide pores, the properties of water far from interfaces and water in the bulk phase are the same, and we can neglect *F*_Vol_. However, water can have a specific structure and energy of intermolecular interactions depending on how far molecules are from interfaces. Thus, the term *F*_Vol_ in eq 3 can be not negligible for microporous materials. The mechanical work *PV* compensates for the energetic and entropic costs of water penetration into the hydrophobic cylinder at the distance *L* from the cylinder mouth, and in the general case
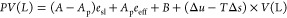
6where Δ*u* and Δ*s* are the excess energy and entropy densities for water in the cylinder not contacting with the surfaces, respectively. The Δ*s* term, a part of which is configurational entropy,^42^ shifts the intrusion pressure upon the constant value, making the pressure positive even for a system with zero energetic cost. Depending on a pore’s geometrical parameters, water energy can be less (Δ*u* < 0) or larger (Δ*u* > 0) than the water energy in the bulk phase, and the intrusion process can be exothermic or endothermic, respectively. The *B*/Δ*V* term is negligibly small at large *V*.

For illustrative purposes, we calculated the sum of the first two terms (eq 6) to highlight the hydrophobic effect of surfaces. The energy cost increases with the waterfront proceeding along the length of the tube (*L*) in the direction of filling. We used simple geometrical formulas (eq S1) to calculate contact areas *A* and *A*_p_ for the growing water cluster in the cylinder (Figure S4).

The following parameters were adopted for numerical calculations: the radius of the cylinder is 6.5 Å; the radius of the side pore is 2.8 Å; and the distance between two pores in a row is 11.6 Å. These approximately correspond to the geometrical parameters of the ITT-type nanotube.

According to the molecular dynamics (MD) simulation results, the intrusion pressure for the nanoparticle with closed pores is 80 MPa. It implies that the intrusion is barrierless at this pressure and *PV* = *Ae*_sl_; thus, one obtains *e*_sl_ = 26 mJ/m^2^. Taking from MD simulations *P*_intr_ = 60 MPa for the nanoparticle with open pores, now, it is easy to calculate effective surface energy, *e*_eff_ = 5.5 mJ/m^2^. We should be careful comparing the macroscopic model with the results of MD simulations when 0–2 water molecules are in each 10MR pore. Nevertheless, the value of *e*_eff_ is reasonable—much smaller than the surface tension of the typical water models,^46^ which are 50–60 mJ/m^2^.

**Figure 7:**
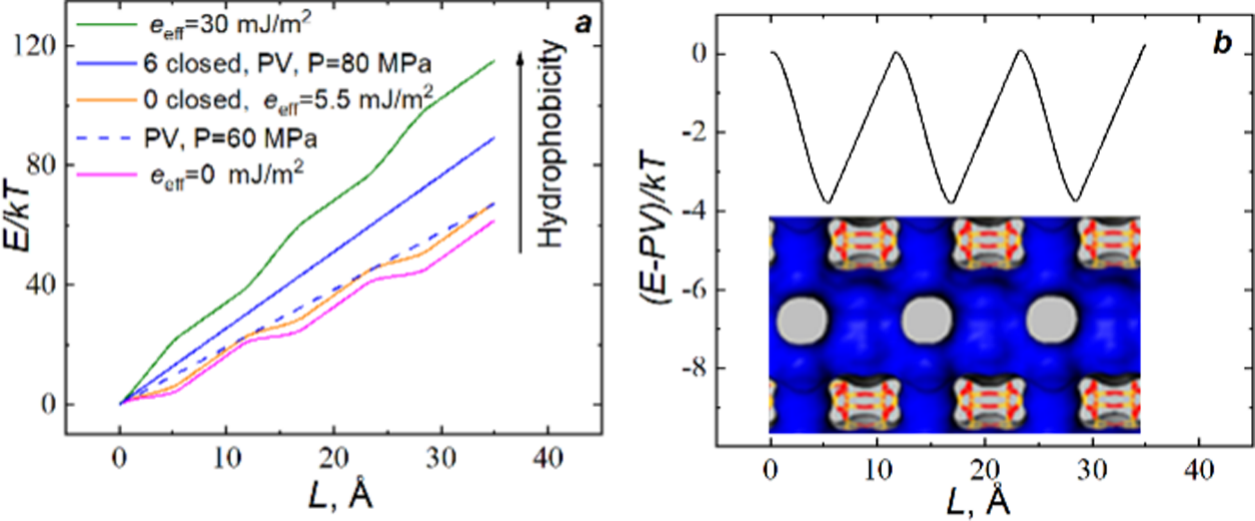
(a) Energetic cost of water penetration in ITT nanoparticles according to the theoretical model. *L* is the length along with the cylinder. Additional curves are calculated for more hydrophilic (*e*_eff_ = 0 mJ/m^2^) and more hydrophobic (*e*_eff_ = 30 mJ/m^2^) side pores. (b) Energy wells for the “0 closed rows” nanoparticle at *P* = 60 MPa corresponding to the positions of 10MR in the ITT-type zeolite presented by Connolly surface.

The results of the calculations are presented in Figure 7. For the cylinder with closed pores, the energetic cost linearly increases with the length of the water column in the cylinder (the blue line). In the case of the cylinder with open pores, the orange curve oscillates along the growing dotted blue line. We varied the hydrophobicity of pores by changing *e*_eff_. If *e*_eff_ < *e*_sl_, the intrusion pressure is smaller than the pressure for the solid cylinder. Moreover, hydrophilic pores locally decrease the free energy of water in the cylinder. Figure 7b highlights this stabilizing effect of open pores. We subtracted the trend line (*PV*) to make the presented plot and demonstrated a local decrease in free energy. The depth of the energy well is about 4 kT. Thus, very simplified considerations show an anchoring role of water in lateral pores. In the case of *e*_eff_ > *e*_sl_, pores are more hydrophobic than the surface, and intrusion pressure is higher than for the solid cylinder. If water spontaneously leaves pores, *e*_eff_ increases, and water in the cylinder can occur in the metastable state, anteceding spontaneous water ejection. This process we observed when drying pores provoked extrusion (Figures 5 and 6).

These considerations regarding the cylinder with water inside it correspond mostly to bulk channels in original ITT zeolite. In the case of tubes immersed in water, we have to consider additional interactions with surrounding water through the silica wall of the channel. The attractive long-range water–water interactions decrease the system’s energy, and the effect is more pronounced for water molecules near the inner surface of the channel. Thus, we can rewrite eq 6 for channels without lateral pores
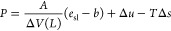
7where *b* is the term corresponding to additional attractive interactions of water molecules in the nanotube with the surrounding water. The same additional term appears in eqs 5 and 6, if we consider nanotubes with side holes immersed in water. As a result, the water intrusion pressure for nanotubes is lower than in bulk channels of the ITT nanoparticles observed in simulations (Figure 2). This explains the avalanche mechanism of wetting a crystal.^35^ The penetration starts from the side and corner channels and propagates into the bulk of the crystal.

The slopes of curves presented in Figure 7a demonstrate an increase in intrusion pressure with an increase in hydrophobicity of the pores. Water in the lateral pores of ITT channels made them more hydrophilic. According to eq 4, *e*_eff_ depends on the hydrophobicity of pores and the fraction of wetted pores. The closing of pores increases the hydrophobicity of materials. Thus, the lowest intrusion pressure is observed for the nanotube with open pores along the surface of the tube with the highest for the tube without these pores (Figure 2a).

For a cylinder without holes,
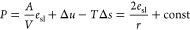
8Thus, eq 8 predicts a linear increase in intrusion pressure with 1/*r* as eq 1, proposed for macroscopic and mesoscopic materials. However, the density of excess free energy can depend on pore radius in the case of microporous materials. We may conclude that our simplified theoretical consideration does not contradict the molecular simulations and rationalizes the MD results.

### Hydrogen Bonding

**Figure 8:**
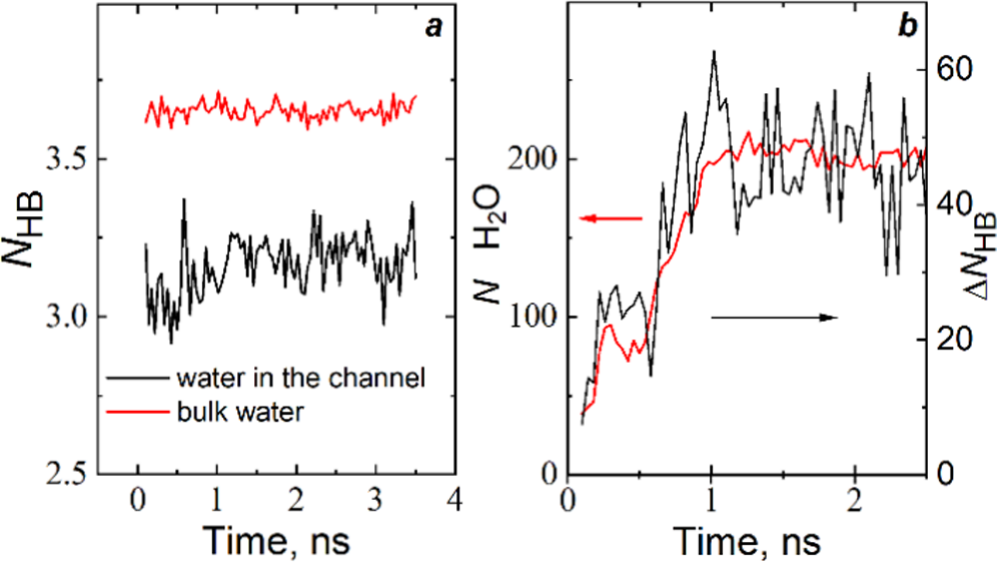
(a) Average number of hydrogen bonds per molecule in bulk water and water in the central channel for the “6 closed rows” nanoparticle at *P* = 100 MPa. (b) Number of broken H-bonds for water in the channel (black line, right *y*-axis) and the number of water molecules in the channel (red line, left *y*-axis).

Additional support for the presented theoretical calculations was obtained by analyzing the statistics of hydrogen bonds formed between water molecules. We applied a simple geometrical criterion, considering molecules as bonded if *r*_OO_< 3.3 Å and *r*_OH_ < 2.41 Å.^47^ As expected, the average number of the H–bonds per water molecule (*N*_HB_) in the hydrophobic channel is lower than in bulk water. In all cases, we observed plots similar to those presented in Figure 8 for the “6 closed rows” nanoparticle at *P* = 100 MPa. The kinetic curves demonstrate fluctuation in the average numbers *N*_HB_ with time. The absence of a sizeable increasing trend in *N*_HB_ for molecules in the two growing water clusters in the channel means a minor role of the “menisci”, which disappear when the clusters coalesce. The number of newly formed bonds is small with respect to the number of bonds in clusters.

The growth of the clusters demands energy to break a part of H-bonds. We have calculated the number of broken bonds according to the formula
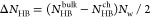
9where *N*_HB_^bulk^ and *N*_HB_^ch^ are the average numbers of H-bonds per molecule in the bulk water and water in the central 18MR channel, respectively. *N*_w_ is the number of water molecules in the channel. The results are presented in Figure 8b. The numbers of broken bonds significantly fluctuate because they are obtained from the two fluctuating terms. However, the plot demonstrates the increase in the energetic cost during the first nanosecond of water penetration. With the growth of clusters entering the channel, water molecules lose a part of H-bonds. After the coalescence of the two clusters, the curve reaches a plateau.

### Prediction of Water Intrusion Pressures for PSZs

**Figure 9:**
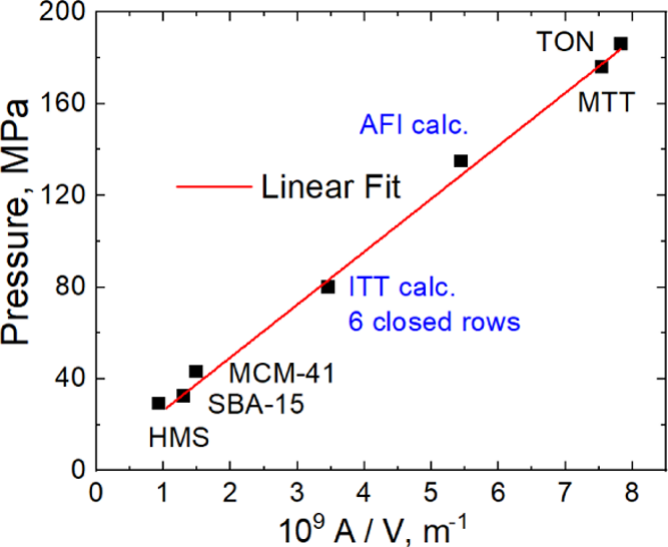
Correlations of intrusion pressures with the ratios of accessible surface to free volume for mesoporous materials^41^ and 10MR TON^49^ and MTT-type^50^ PSZs. Calculated data for AFI and ITT were taken from the previous publication^42^ and present work, respectively.

The proposed theoretical method can be used to estimate the intrusion pressure in porous materials. According to eq 8, intrusion pressure is defined by the ratio of accessible surface to free volume of the pore. To calculate the intrusion pressure, we propose to use Connolly^48^ free volume *V* and accessible surface *A* at a radius of the probe particle of 2 Å.^42^Figure 9 demonstrates a comparison of experimental and calculated intrusion pressures: for three mesoporous silica materials grafted with octylsilanes (MCM-41, SBA-15, and HMS);^41^ zeolites with the a 1D system of 10MR channels (TON, MTT); and calculated pressures for AFI-^42^ and ITT-type zeolite with closed 10MR pores. For mesoporous materials, *A*/*V* values were calculated using the published pore radii.^41^ The formation of silanol defects during intrusion makes AFI-type zeolite less hydrophobic.^42^ Thus, we used calculated pressure instead of the experimental one. In Figure 9, the presented values of pressures are close to the fitting line, which demonstrates the applicability of eq 8 for some mesoporous and microporous materials.

We cannot predict intrusion pressure for zeolites based on only the theoretical considerations of primitive models because actual materials have a different topology of frameworks, size, and shape of pores and defects of crystal structures. The structure and density of water in pores, energy, and entropy depend on these parameters.

Traditionally, according to eq 1, one attempts to correlate *P*_intr_ with a reverse radius of a pore opening (1/*r*). However, the correlation is poor.^31,32^ Therefore, we propose correlating experimental data with *A*/*V* (eq 8). Figure 9 shows that the correlation is observed for both meso- and microporous materials. To check the hypothesis, we took cif files from the Database of Zeolite Structures as they were.^10^ These are ideal structures, while actual materials have slightly different parameters of unit cells.

**Figure 10:**
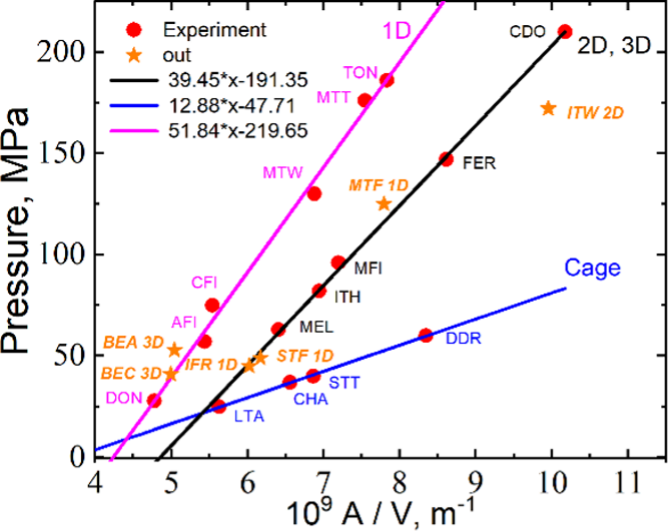
Correlations of intrusion pressures with the ratios of Connolly accessible surface to free volume calculated for pure silica zeolites.

The results of the calculations are presented in Figure 10, where we plot three straight lines for different topology classes of PSZs corresponding to 1D, two-dimensional (2D), 3D, and cage structures of pores. Unexpectedly, only one PSZ is out of linear correlations, namely ITW. This plot supports our conclusions about the role of the topology in the intrusion process. At the same *A*/*V*, intrusion pressure is higher for the 1D topology of pore systems than for 2D or 3D. Only six PSZs from 22 are out of the adopted classification, but five points lie on these straight lines. Thus, the topology is one of the main parameters controlling intrusion pressure.

As a rule, all PSZs have structure defects: the broken bonds in −Si–O–Si– chains terminated with silanol (−Si–OH) and siloxy (−Si–O^–^) groups.^51−53^ These groups significantly decrease the hydrophobicity of zeolites and thus the intrusion pressure. The defects are formed during water intrusion, and for some PSZs, the pressure significantly decreases with the number of intrusion/extrusion cycles. However, the stability of zeolites in water is different and depends on many factors. Degradation of the materials can be observed by many experimental methods with a change of X-ray diffraction patterns, scanning electron microscopy (SEM) images, ^29^Si MAS and ^1^H–^29^Si CPMAS NMR spectra, *etc.*(54) It was demonstrated that TON and MTT PSZs have chemically stable structures.^49,50^ Meanwhile, STF,^50^ ITW,^55^ IFR,^56^ -SVR (SSZ-74),^51^ and some other PSZs have low stability. The presence of defects is one of the reasons why the slopes of the straight lines presented in Figures 9 and 10 for materials with the 1D channel system are different.

We can suppose that the stability decreases with decreasing *A*/*V*.^42^ For ITQ-12 (ITW) and Mu-26 (STF), experiments clearly point to breaking some siloxane bridges.^55,57^ ITW-type zeolite has a peculiar structure, including narrow flattened cages connected through 8MR windows. The topology has the features of 1D and cage structures. We may suppose that the formed water structure does not fit the inner shape of 2D channels. The pore volume obtained from N_2_ adsorption is 0.12 cm^3^/g, but the zeolite contains only 0.047 cm^3^/g of water at high pressure.^55^ Silanol groups can clog some pores, decreasing the accessible volume.^51^

We have proposed an empirical approach, and it was unexpected that only one PZS was out of fitting lines. The specific water structure in pores, the energy–entropy term in eq 8, is not directly counted in these correlations. We think that zeolites are joined in groups due to specific water structures in pores and the topology of the zeolite. Additional systematic investigations, including simulations and analyses of synthesized zeolites, need to clarify the situation.

Data for ITQ-4 (IFR) PSZ were discussed previously.^42^ Calculations show a significantly higher value of *P*_intr_ in IFR than experimental ones. Zeolite *BEA is partially disordered, and BEC is one of the polymorphs of the family.^52,58^ In these zeolites, the system of channels presents the 3D network of intersecting cylinders (12MR pore opening). A regular straight channel system may be closer to zeolites with a 1D topology.

We may summarize the possible problems of our PSZ classification: quality of synthesized zeolites; variations of topologies in one class of zeolites; simplicity of the theoretical model; different energy–entropy terms for zeolites (eq 8); and not optimized or not experimental parameters of unit cells for which we have calculated *A*/*V*. Even the width of walls between pores can be valuable due to the difference in the strength of water–water and water–silica interactions. Nevertheless, correlations with *r*^2^ = 0.999 for cages, 2D–3D, and with *r*^2^ = 0.993 for 1D structures are surprising.

The mechanical energy of intrusion is calculated by *E*_intr_*= P*_intr_*V*, where *V* is the intruded volume of water. The accessible volume correlates with the intruded volume, but they are different. The density of water in pores depends on the pore geometry and topology. For simplicity, we used the accessible volume of pores as the intruded volume. Our calculations of mechanical energy are performed according to the equations
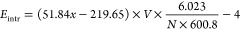
10
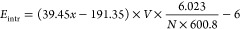
11where *x* = 10 *A*/*V* is measured in Å and *N* is the number of Si atoms in the unit cell. These equations are especially presented in a simplified form for easier application. Coefficients of fitting lines are shown in Figure 10.

**Figure 11:**
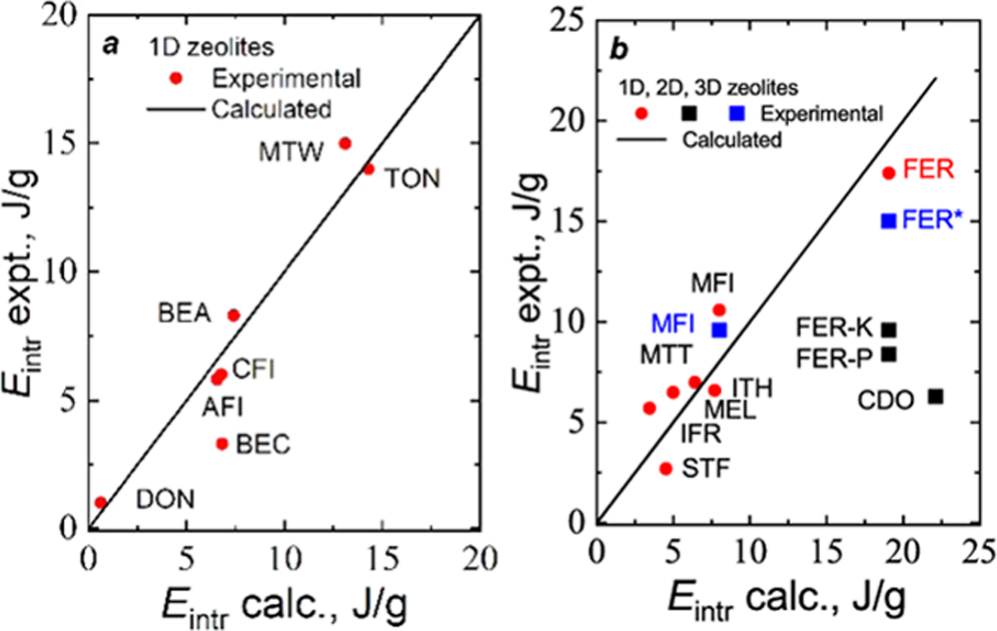
Correlations of experimental and calculated intrusion energies: (a) for PSZs with the 1D system of channels and (b) for PSZs from the middle branch of Figure 10. The experimental data were taken from several sources.^15,16,31,32,60−62^

The correlations between experimental and calculated data are presented in Figure 11. The cases where more than one source is available allow discussion on the possible reasons behind some discrepancies between experimental and predicted data. For silicalite-1 (MFI), the results of two experiments are available: 10.6 J/g^32^ and 9.6 J/g.^59^ The difference of 10% is not too significant. However, for FER, we have four data, and they are very different: 17.4,^60^ 15.0,^32^ 9.6, and 8.4 J/g.^61^ Energy is a product of pressure and intruded water volume. The main difference in data is observed for the volume. Clogging pores due to structural defects can be the main factor contracting the accessible volume.

Several factors may influence experimental results. The first is the quality of zeolite samples. The FER-P and FER-K crystals were synthesized using two protocols, and the adsorbed water volume differed.^61^ Another source of possible mistakes may be connected with raw data treatment. Results of grand canonical Monte Carlo simulations are close to the two first values.^62^ Depending on the pressure, 13–15 water molecules occupy each crystal unit cell. Probable positions of molecules in pores are determined.^60^ Another source of possible inconsistencies may be connected with raw data treatment. CDO-type zeolite is far from the proposed correlation line. CDO-type zeolite is far from the proposed correlation line. The intrusion curve for this zeolite looks very unusual. Intrusion starts at 150 MPa and finishes at 300 MPa.^61^ Our empirical correlation corresponds to two experimental data^32,60,62^ for FER and does not correspond to the other three data (FER-K, FER-P, CDO).^61^ Thus, using established correlation, one can estimate possible intrusion pressure and energy and select more confident values from the existing database.

**Figure 12:**
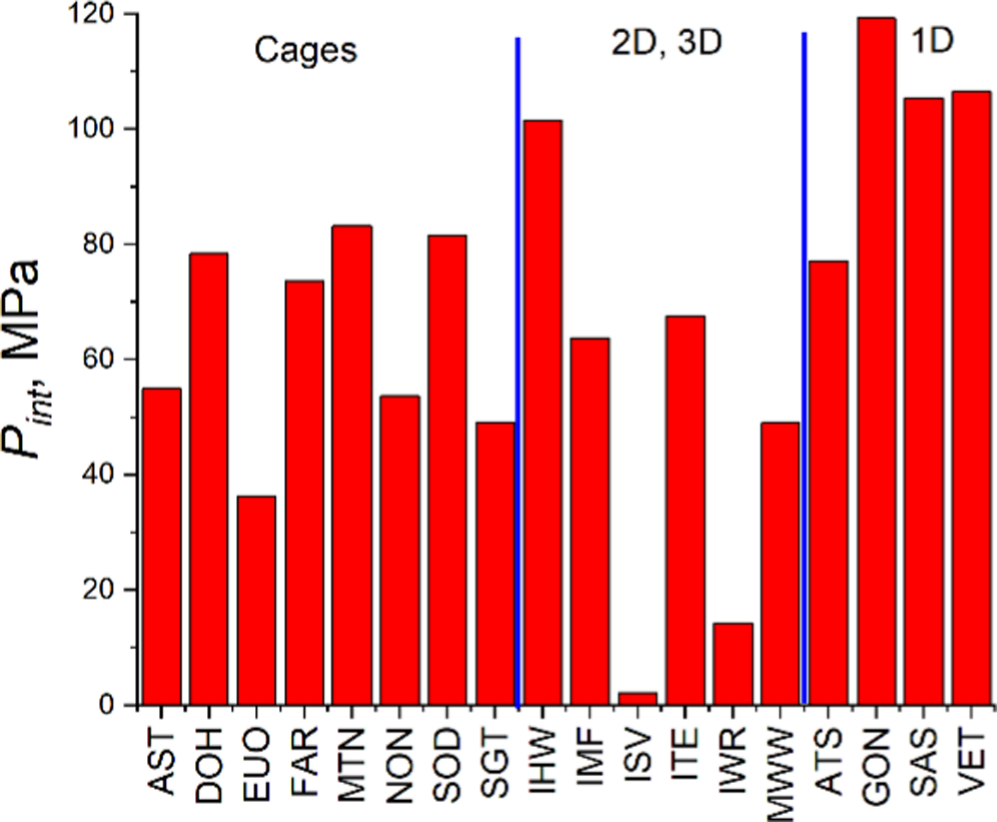
Predicted intrusion pressures for pure silica zeolites.

The zeolite database^10^ contains the topologies of PSZs for which experimental intrusion pressures are unknown. The main advantage of the proposed approach is the minimal demands in input data. We need to know only cif files to calculate *A*/*V*. The results for 18 topologies are presented in Figure 12. We may expect that predictions can be incorrect for some zeolites, as in the case of any extrapolation not all factors are counted, but it gives us a set of systems for further investigations. We suppose that DOH, MTN, SOD, IHW, GON, SAS, and VET topologies can demonstrate high intrusion pressures. The PSZs with the ISV and IWR topology would be fascinating to look at in terms of low-pressure applications. We will simulate some of these zeolite–water systems to check the predictions in our future research.

## Conclusions

Nano-objects based on the ITT-type zeolite topology were chosen to investigate the role of secondary porosity and topology in the hydrophobicity of microporous materials. The zeolite has a system of extra-large (18MR), straight channels interconnected by lateral pores (10MR). It makes this microporous crystal resemble mesoporous amorphous materials. Two objects immersed in water were investigated: pure silica nanoparticles and nanotubes tailored from the ITT crystal. We studied the effects of closing 10MR pores on the water–zeolite system behavior in a broad range of hydrostatic pressures using molecular dynamics simulations. Intrusion pressures characterized the hydrophobicity of the microporous materials.

We demonstrated that a change of secondary porosity alters the hydrophobicity of these pure silica nano-objects. Both water intrusion/extrusion pressures increase with a decreasing amount of open 10MR pores. Similar results were obtained previously for silicalite-1 (MFI) PSZ, where pores have other shapes and lengths.^35^

From a macroscopic point of view, wetting hydrophobic narrow pores before larger pores is impossible. However, simulations of nanotubes demonstrated that water molecules appear in short 10MR pores before the water enters 18MR channels. A part of these pores contain some water molecules at any moment. We observed mutual stabilization of water clusters in pores during water protruding into the nano-objects. The number of molecules in both pores and channels increases progressively during the intrusion process. We obtained the explanation of the proposed molecular mechanism of ITT-type crystal wetting.^35^ Due to the higher hydrophobicity of nanotubes, water initially penetrates side channels, which are all-time exposed to water, and then propagates to the bulk of the crystal.

Water in lateral pores plays the anchoring role for water in channels during extrusion. Before expulsion from channels, the number of molecules in the 10MR pores decreases drastically, destabilizing water clusters in the channel. The metastable cluster loses connectivity, and molecules start to move fast to the channel mouths. The process resembles a breaking over-stressed spring.

The systems demonstrate intrusion–extrusion hysteresis. We must highlight that the duration of extrusion can depend on the height of a free energy barrier between metastable filled and empty states. In MD simulations, the process develops for only several nanoseconds, and extrusion may occur at a lower pressure than in an actual experimental situation. At the same time, the hysteresis is absent for some systems, and they demonstrate the “spring” behavior in both computer simulations and experiments. We carried out intrusion/extrusion simulations under the same conditions and observed the same effect when changing the topology of nano-objects: both pressures increased with the closing of the lateral pores.

In this work, we attempted to use all the available experimental data, which can support the effect of the secondary topology. Moreover, based on theoretical considerations, we rationalized our simulations’ results and proposed to use the ratio of accessible surface area to pores volume (*A*/*V*) as a key parameter, attempting to establish correlations between intrusion pressure and the topology of hydrophobic porous materials. We showed that the correlation does exist for some functionalized mesoporous amorphous materials and pure silica zeolites. Thus, we suppose that topological tuning of hydrophobicity applies to mesoporous materials.

Based on the *A*/*V* ratio, we investigated the existing data of intrusion pressures into pure silica zeolites, found three groups of zeolites with 1D, 2D–3D, and cage topologies, and predicted intrusion pressure and absorbed mechanical energy for the set of hydrophobic zeolites. The results can help choose prospective candidates for further investigation to improve the energetic performance of water–zeolite systems.

Thus, taking into account (i) the results published in our previous paper, where we showed the role of the secondary porosity in ITT- and MFI-type crystals; (ii) the results obtained in the presented work for ITT nanotubes; and (iii) theoretical considerations and the empirical correlations, we can suppose that the established molecular mechanism of wetting/drying can be exploited to design new porous materials with tuned hydrophobicity, which are zeolites, functionalized amorphous silica, and MOFs. The hydrophobicity can be controlled without significantly altering the chemistry and structure of materials by creating/eliminating secondary porosity or changing the ratio of open to closed lateral pores. The design of new materials can be based on the established principle.

## Methods

The size of the system, number of particles, and complexity of their interactions essentially limit computer simulations of mesoporous amorphous materials and nanotubes. Even in the case of zeolites, this is a challenging task due to the enormous demands in computational facilities. The task can be solved for simplified models, such as the Kiselev model^38^ traditionally used to calculate adsorption isotherms and computational simulations.^63,64^ It is based on two assumptions: a rigid framework and neglecting van der Waals interactions with silicons. Simplification of force fields reduces the computational cost significantly, allowing the simulation of large systems with the same computational resources.

A detailed explanation of the used methods and validations of results are presented in the previous paper.^35^ The choice of force fields, the role of partial electrical charges in atoms, and the flexibility of frameworks are discussed there. We applied the same simulation method adopted for ITT nanoparticles immersed in water to be consistent. The calculations are based on the Bushuev–Sastre force field, showing good results for PSZ and zeolite–SDA–water systems.^42,51,65^ This force field is the modified ClayFF.^66^ The parameters of Lennard-Jones interactions are ε = 2.238 J/mol, σ = 3.234 Å for Si–O_w_ (O_w_ is oxygen of water) and ε = 650.2 J/mol, σ = 3.1655 Å for O–O_w_ atoms. All Si and O atoms were fixed in their crystallographic positions to save the zeolite structure during the simulations. DL_POLY version 4.09 and 4.10^67^ were used for molecular dynamics simulations of the porous materials immersed in water. Calculations were performed in an NPT ensemble. An isotropic thermostat and barostat with Melchionna modification^68^ of the Nosé–Hoover algorithm with a relaxation time of 1 and 5 ps, respectively, were applied to calculate systems at *T* = 300 K and a set of hydrostatic pressures. The flexible SPC/Fw^69^ water model was used.^69^

Short-range van der Waals and electrostatic interactions were limited by a spherical cut-off radius of 9 Å, as recommended in the original article.^69^ The smooth particle mesh Ewald (SPME) method was employed to calculate the long-range part of electrostatic interactions of water molecules. Equations of motion were solved numerically by the velocity Verlet integrator with the timestep of 1 fs. Intrusion/extrusions were observed due to step-by-step increasing/decreasing pressure starting from empty/filled porous materials. DL_POLY files containing all information about the simulation method and force fields are presented in the Supporting Information.

## Supporting Information Available

Water intrusion into the nanotube with open 10MR pores (AVI)
Water intrusion into the nanotube with closed 10MR pores (AVI)
Water extrusion from the nanotube with open 10MR pores (AVI)
Open tube at 60 MPa data (ZIP)
Kinetics of water extrusion from 18MR channels of nanoparticles with open pores; snapshots with the timestep of 40 ps of the central channel of the nanoparticle with closed 10MR pores during the extrusion process; and legend for video files (PDF)

## Supplementary Material

am2c06039_si_001.avi

am2c06039_si_002.avi

am2c06039_si_003.avi
