# Supplementary material for: Effect of the Topology on Wetting and Drying of Hydrophobic Porous Materials
Source: ACS Appl Mater Interfaces. 2022 Jun 22;14(26):30067–79. doi: 10.1021/acsami.2c06039 (PMC9264313; doi:10.1021/acsami.2c06039)
Supplement: Supplementary file 5 — am2c06039_si_005.pdf [file am2c06039_si_005.pdf]

SUPPORTING INFORMATION

Effect of the topology on wetting and drying of hydrophobic porous materials

Yuriy G. Bushuev[a]*, Yaroslav Grosu[b], Mirosław A. Chorążewski[a], Simone Meloni[c]

[a]*Institute of Chemistry, University of Silesia in Katowice, Szkolna 9 street, 40-006 Katowice, Poland*

[b]*Centre for Cooperative Research on Alternative Energies (CIC energiGUNE), Basque Research and Technology Alliance (BRTA), Alava Technology Park, Albert Einstein 48, 01510 Vitoria-Gasteiz, Spain*

[c]*Dipartimento di Scienze Chimiche, Farmaceutiche ed Agrarie (DOCPAS), Università degli Studi di Ferrara (Unife), Via Luigi Borsari 46, I-44121, Ferrara, Italy*

Email: yuriy.bushuev@us.edu.pl

Kinetics of water extrusion from 4x4x5 unit cells ITT nanoparticle

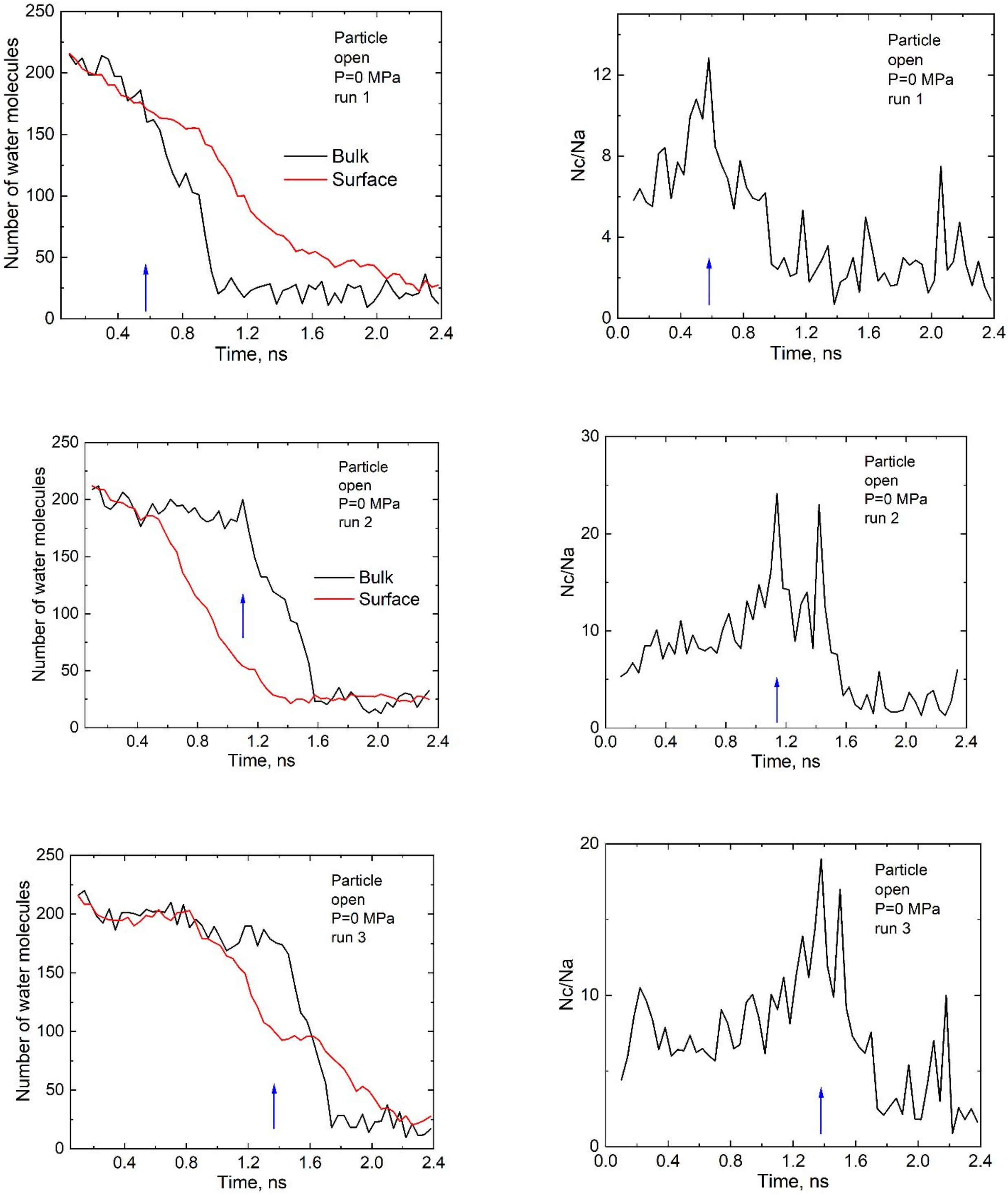

Figure S1. Kinetics of water extrusion from 18MR channels of nanoparticle with open pores. The left panel: number of molecules vs. time: in the bulk (black line) and surface (red line) channels. The right panel: the number of molecules in the central channel (N_c) to those (N_a) in adjoined 10MR windows. The arrows indicate the starting time of extrusion from the bulk channel.

t=0 ps

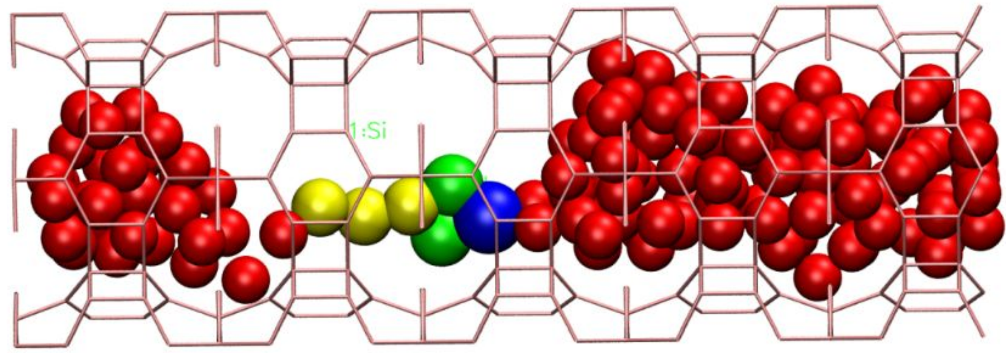

t=40 ps

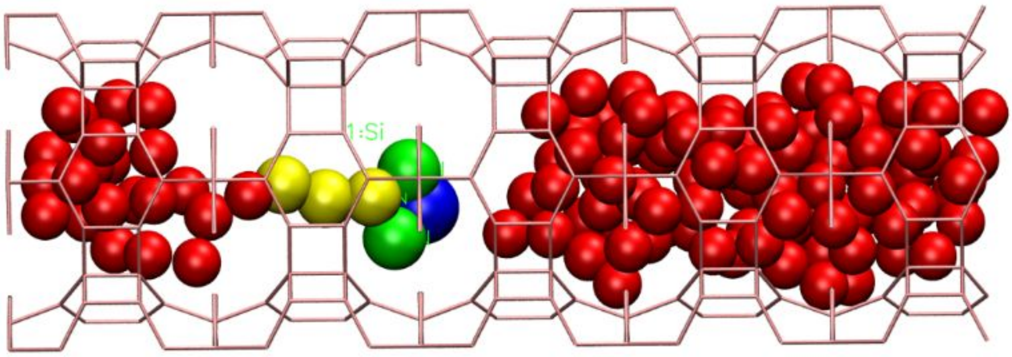

t=80 ps

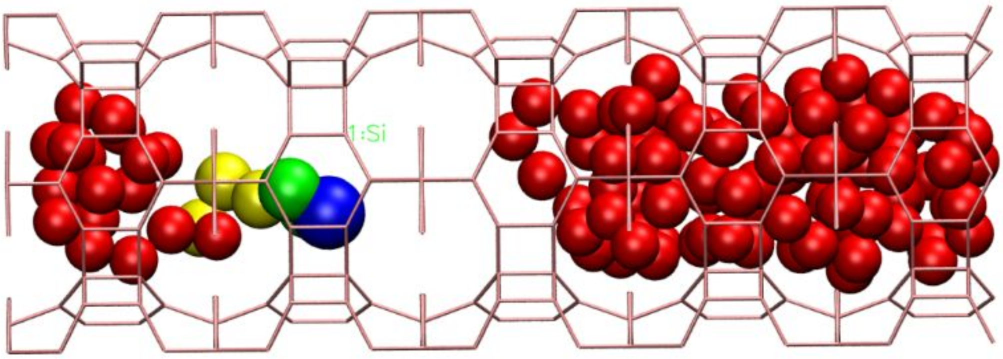

t=120 ps

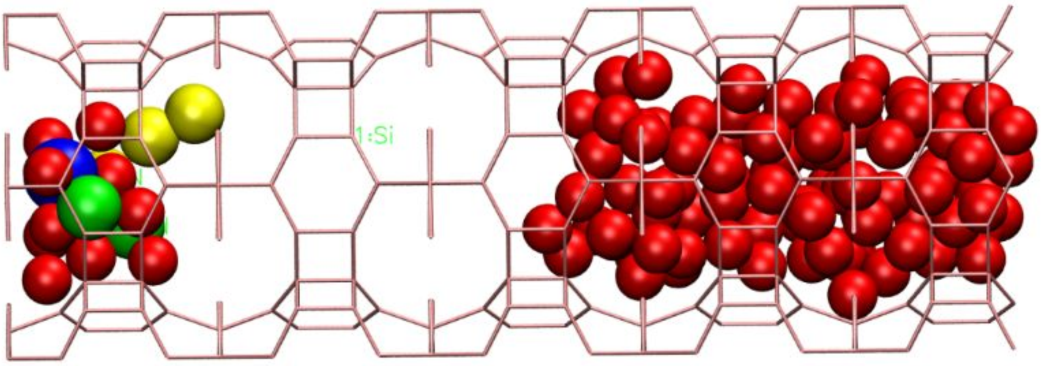

Figure S2. Snapshots with the timestep of 40 ps of the central channel of the nanoparticle with closed 10MR pores during the extrusion process. For clarity, water molecules were coloured in different colours; the framework is shown by sticks connecting Si atoms; water outside the tube is not shown.

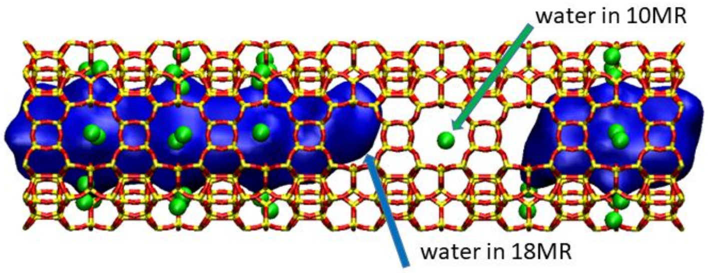

Figure S3. Legend for Movies. The snapshot of the nanotube with open 10MR pores. The water in the 18MR channel of the nanotube is shown as blue surface, in lateral 10MR pores as green spheres; water outside the tube is not shown.

Movies S1 and S2 demonstrate water intrusion into the nanotube with open and closed 10MR pores.

Movies S3 demonstrates water extrusion from the nanotube with open 10MR pores.

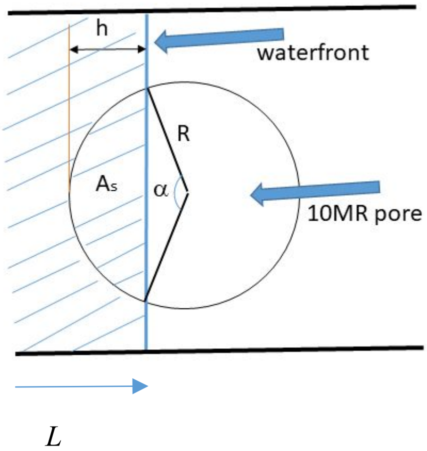

Figure S4. Scheme and equations for calculations of areas.

$$A_s(h) = 0.5 \times R^2(\alpha - \sin\alpha)\,, \qquad \text{where } \alpha = 2\arccos\left(1 - \frac{h}{R}\right). \qquad \text{(S1)}$$
